# Clinical relevance of the STOPP/START criteria in hip fracture patients

**DOI:** 10.1007/s00228-016-2188-9

**Published:** 2017-01-03

**Authors:** Johan Lönnbro, Susanna M. Wallerstedt

**Affiliations:** 1 Department of Internal Medicine, Sahlgrenska University Hospital, Gothenburg, Sweden; 2 Department of Pharmacology, Sahlgrenska Academy, University of Gothenburg, SE-413 90 Gothenburg, Sweden; 3 Department of Clinical Pharmacology, Sahlgrenska University Hospital, Gothenburg, Sweden

**Keywords:** Clinical relevance, Pharmacoepidemiology, Prescribing quality, START criteria, STOPP criteria

## Abstract

**Purpose:**

The aim of this study was to investigate the clinical relevance of potentially inappropriate medications (PIMs), identified by the STOPP criteria, and potential prescribing omissions (PPOs), identified by the START criteria, and to identify predictors for clinically relevant PIMs and PPOs.

**Methods:**

The STOPP and START criteria were applied on the medication lists of 200 older hip fracture patients, consecutively recruited to a randomized controlled study in 2009. For each identified PIM and/or PPO, the clinical relevance was assessed at the individual level, using medical records from both hospital and primary care as well as data collected in the original study.

**Results:**

A total of 555 PIMs/PPOs were identified in 170 (85%) patients (median age: 85 years, 67% female), 298 (54%) of which, in 141 (71%) patients, were assessed as clinically relevant. A greater proportion of PIMs than PPOs were clinically relevant: 71% (95% CI: 66%; 76%) vs. 32% (27%; 38%). A greater proportion of PPOs than PIMs could not be assessed with available information: 38% (32%; 44%) vs. 22% (17%; 27%). Number of drugs and multidose drug dispensing, but not age, sex, cognition, or nursing home residence, were associated with ≥1 clinically relevant PIMs/PPOs.

**Conclusions:**

The present study illustrates that one in two PIMs/PPOs identified by the STOPP/START criteria is clearly clinically relevant, PIMs being clinically relevant to a greater extent than PPOs. Based on available information, the clinical relevance could not be determined in a non-negligible proportion of PIMs/PPOs. Number of drugs and multidose drug dispensing were associated with ≥1 clinically relevant PIMs/PPOs.

## Introduction

Prescribing of drugs is a challenge, particularly in older people who are sensitive to drug effects and often suffer from multiple morbidities. In fact, it is well-known that suboptimal pharmacotherapy is common in older people, such as treatment with inappropriate drugs or dosages, and/or omissions of drugs which the patient would probably benefit from [1–3].

In order to improve the quality of drug treatment, explicit criteria may serve as tools to identify potential problems, as potentially inappropriate medications (PIMs) and potential prescription omissions (PPOs). Some criteria can be applied on the drug list alone, whereas others require clinical information such as diagnoses and laboratory parameters [4–7]. The screening tool of older persons’ potentially inappropriate prescriptions (STOPP) and the screening tool to alert to right treatment (START) criteria belong to the ones requiring clinical information [8]. These criteria have been used in several studies describing drug treatment quality [9, 10]. The original version was developed to be applied in the clinical situation by the attending physician, and provide 65 criteria for potentially inappropriate drugs and 22 criteria for potentially missing drugs, respectively. An updated, more extensive, version has been published [11].

To apply the first version of the STOPP/START criteria has been estimated to require an additional 3 min for the physician-patient consultation [12]. However, even though the additional minutes required may be few under optimal conditions, constraints of time may be an obstacle for implementation [13]. Indeed, as information on medical conditions and medication history within an electronic patient record is not always easily retrieved [14], systematically checking an extensive set of criteria may be time-consuming.

To facilitate for physicians to take advantage of criteria in clinical practice, the first step may be to minimize the extra time needed for the application. This can be achieved by focusing on frequently occurring criteria, easy to assess given available information in the medical records, and often of clinical relevance. However, although experts have often assessed available criteria sets for relevance, credibility, and acceptability at the general level [15, 16], less is known about the extent of their clinical relevance at the individual level.

In addition to easily applicable criteria requiring a minimum of time, physicians may want to know which patients would be most worthwhile to assess a bit further. Indeed, potentially suboptimal drug treatment has been associated with age, sex, and number of drugs [17]. However, less is known on patient characteristics associated with clinically relevant prescribing problems, a question of superior interest to the attending physician.

The aim of this study was to investigate the clinical relevance of PIMs and PPOs identified by the STOPP/START criteria, and to identify predictors for clinically relevant PIMs/PPOs.

## Methods

The present study was performed within a cohort of older hip fracture patients, consecutively recruited to a randomized controlled study in the departments of orthopedics, geriatrics, and medicine at Sahlgrenska University Hospital in 2009 [18]. Inclusion criteria in the original study were patients, ≥65 years of age, who had undergone surgery for a hip fracture at Sahlgrenska University Hospital, were residing in the Gothenburg area, and provided informed consent. In all, 200 out of 253 patients undergoing hip fracture surgery during the inclusion period were included in the original study; 23 declined participation, 14 did not fulfill the other inclusion criteria, 10 were deceased before inclusion, and six were excluded because of other reasons.

In the original study, the medication list at study entry (admission to hospital) was determined. When a patient could not report satisfactorily on his/her medications, the Swedish Register of Dispensed Drugs (Läkemedelsförteckningen) was consulted, holding information available in clinical practice for the attending physician on prescribed drugs purchased from any Swedish pharmacy during the preceding 15 months. Drugs used regularly and as needed were included. Drugs for external use were included only if having potential systemic effects. Thus, topical medications, tear substitutes, and dental fluoride preparations were excluded.

In the present study, the STOPP and START criteria were applied on the medication list determined in the original study, identifying PIMs and PPOs. The clinical relevance of each PIM/PPO was assessed at the individual level. If the expected benefit of a particular medication was judged to outweigh the potential harm, such as an antipsychotic drug in a patient with schizophrenia, the PIM was assessed as not clinically relevant. Similarly, if there was a clinical reason not to treat the patient with the drug, such as an adverse drug reaction or a contraindication, the PPO was assessed as not clinically relevant. When the information available was not sufficient to determine the clinical relevance, the PIM/PPO was categorized as not assessable.

The assessments were independently performed by one general practitioner and one geriatrician in 2012–2013. They were based on (1) medical records from both hospital and primary care and (2) previously collected data including information on risk of falls, cognition, residence, and glomerular filtration rate. The latter, estimated with the Cockcroft-Gault equation, was dichotomized as either ≥50 or <50 ml/min to fit the STOPP and START criteria. In a final consensus discussion, the two specialist physicians reached agreement on identified PIMs/PPOs, and the clinical relevance of these.

### Statistics

All statistical analyses were performed with SPSS (IBM SPSS Statistics for Windows, Version 17.0, Armonk, NY). We used the Mann Whitney and the chi-square tests for comparisons of characteristics between patients with and without ≥1 clinically relevant PIMs/PPOs, and 95% confidence intervals (CI) were calculated for proportions. Kappa statistics was used to assess inter-rater agreement. Logistic regression was performed to obtain odds ratios (and 95% CI) for ≥1 PIMs/PPOs, as well as ≥1 clinically relevant PIMs/PPOs. In the main analysis, due to the fact that we wanted to identify quickly adoptable criteria, we chose to categorize not assessable PIMs/PPOs as not clinically relevant. A sensitivity analysis was performed where not assessable PIMs/PPOs were categorized as clinically relevant. Covariates included in the analysis were age, sex, cognition (defined as impaired or not), residence (defined as nursing home or not; nursing home residence reflecting that the patient needed help with daily living activities), multidose drug dispensing (defined as having ≥1 drugs prescribed via this system; associated with quality of drug treatment in prior studies [19–21]), and number of drugs (a proxy for burden of disease [22]).

## Results

Characteristics of patients by results on STOPP/START criteria are presented in Table 1. Summarized, the patients had a mean age of 84.5 years, ranging from 65 to 98 years, and 133 (67%) were women. The mean number of drugs in the medication list was 7.2 ± 3.9 (range 0–21). Multidose drug dispensing, was consistently more common in patients with PIMs/PPOs, irrespective of their being clinically relevant or not. These patients also had more drugs in their medication list.

**Table 1:** Characteristics of patients according to presence of ≥1 potentially inappropriate medications (PIMs) and/or ≥1 potential prescription omissions (PPOs), identified by the STOPP and START criteria, respectively

		≥1 PIMs/PPOs			≥1 clinically relevant PIMs/PPOs		
		Yes	No		Yes	No	
		*n* = 170	*n* = 30	*P* value	*n* = 141	*n* = 59	*P* value
Age	Mean ± SD	85.1 ± 6.7	81.1 ± 8.3		84.8 ± 6.8	83.7 ± 7.6	
	Median (range)	85 (65–98)	80 (65–98)	0.009	85 (65–98)	85 (65–98)	0.37
Female sex		112 (66)	21 (70)	0.66	92 (65)	41 (69)	0.56
Multidose drug dispensing		97 (57)	3 (10)	<0.0001	86 (61)	14 (24)	<0.0001
Impaired cognition		84 (49)	6 (20)	0.003	69 (49)	21 (36)	0.084
Residing in nursing home		56 (33)	4 (13)	0.031	47 (33)	13 (22)	0.11
Number of drugs	Mean ± SD	7.7 ± 3.7	4.1 ± 3.7		8.2 ± 3.6	4.8 ± 3.6	
	Median (range)	7 (0–21)	3 (0–15)	<0.0001	8 (2–21)	4 (0–15)	<0.0001

Values are presented as number of patients (percentage) if not stated otherwise

*SD* standard deviation, *STOPP* screening tool of older person’s potentially inappropriate prescription, *START* screening tool to alert to right treatment

A total of 555 PIMs/PPOs were identified in 170 (85%) patients; 298 (54%) of which, in 141 (71%) patients, were assessed as clinically relevant. The inter-rater agreement was moderate (kappa 0.52). A greater proportion of PIMs than PPOs was clinically relevant: 217 in 305 (71% (95% CI: 66%; 76%)) vs. 81 in 250 (32% (27%; 38%)). The median number of clinically relevant PIMs and PPOs per patient was 1 (mean 1.09, range 0–6) and 0 (mean 0.41, range 0–3), respectively.

In all, 160 (29%) PIMs/PPOs were not assessable. A greater proportion of PPOs than PIMs could not be determined with available information: 94 (38% (32%; 44%)) vs. 66 (22% (17%; 27%).

For six (9.2%) out of 65 STOPP and two (9.1%) out of 22 START criteria, ≥10 (5%) patients had a clinically relevant PIM and PPO, respectively (Table 2). For 37 (57%) STOPP and 17 (77%) START criteria, ≥1 (≥0.5%) patients had a clinically relevant PIM and PPO, respectively.

**Table 2:** Potentially inappropriate medications (PIMs) and potential prescription omissions (PPOs), identified by the STOPP and START criteria, respectively, identified in ≥5% of the study cohort (*n* ≥ 10)

Description		PIM/PPO*	Clinically relevant PIM/PPO**	Not clinically relevant PIM/PPO**	Not assessable PIM/PPO**
Benzodiazepines in those prone to falls	STOPP	76 (38)	47 (62)	0 (0)	29 (38)
Osteoporosis and no calcium and vitamin D supplementation	START	61 (31)	10 (16)	4 (7)	47 (77)
Chronic atrial fibrillation without warfarin	START	29 (155)	7 (24)	14 (48)	8 (28)
Aspirin with no history of coronary, cerebral or peripheral arterial symptoms, or occlusive arterial event	STOPP	27 (14)	13 (48)	12 (44)	2 (7.4)
Aspirin at dose >150 mg day	STOPP	24 (12)	23 (96)	1 (4)	0 (0)
Loop diuretic for dependent ankle edema only i.e., no clinical signs of heart failure	STOPP	24 (12)	20 (83)	0 (0)	4 (17)
Atherosclerotic disease with sinus rhythm without aspirin or clopidogrel	START	21 (11)	9 (43)	8 (38)	4 (19)
Chronic heart failure without ACE inhibitor	START	20 (10)	5 (25)	10 (50)	5 (25)
Diabetes mellitus and ≥1 coexisting major cardiovascular risk factor without a statin	START	19 (9.5)	4 (21)	11 (58)	4 (21)
Neuroleptic drugs in those prone to falls	STOPP	17 (8.5)	11 (65)	0 (0)	6 (35)
Chronic stable angina and no beta-blocker	START	17 (8.5)	10 (59)	3 (16)	4 (24)
Vascular disease and a life expectancy of >5 years without a statin	START	16 (8)	9 (56)	2 (13)	5 (31)
Long-term opiates in those with recurrent falls	STOPP	16 (8)	9 (56)	0 (0)	7 (44)
Prior acute myocardial infarction and no ACE inhibitor	START	15 (7.5)	3 (20)	7 (44)	5 (33)
Long-term long-acting benzodiazepines	STOPP	13 (6.5)	13 (100)	0 (0)	0 (0)
Vasodilator drugs known to cause hypotension in those with persistent postural hypotension	STOPP	11 (5.5)	9 (82)	1 (9)	1 (9.1)
Diabetes mellitus and ≥1 coexisting major cardiovascular risk factor without antiplatelet therapy	START	11 (5.5)	2 (18)	7 (64)	2 (18)
Maintained oral corticosteroid therapy without bisphosphonates	START	10 (5)	5 (50)	3 (30)	2 (20)

Values are presented as number of patients (*percentage of all patients; **percentage of patients with PIM/PPO)

*ACE* angiotensin converting enzyme, *STOPP* screening tool of older person’s potentially inappropriate prescription, *START* screening tool to alert to right treatment

The most frequently occurring PIMs were benzodiazepines in those prone to falls, and aspirin either without cardiovascular disease or at a dose >150 mg/day. The PIMs long-acting benzodiazepines and aspirin at a dose >150 mg/day were clinically relevant in all but one patient. As for loop diuretics without clinical signs of heart failure and for bensodiazepines, no PIM was assessed as not clinically relevant. Although not frequently occurring (5.5%), the PIM vasodilator drugs in patients with postural hypotension were clinically relevant in 82% of the cases. Among patients prone to falls, the clinical relevance of the frequent PIMs concerning benzodiazepines, neuroleptics, and opiates could not be determined in 35–44% of the cases.

The most frequently occurring PPOs were osteoporosis without calcium and vitamin D supplementation, and chronic atrial fibrillation without warfarin; 16% and 24% of which, respectively, were assessed as clinically relevant. Clinical relevance could not be determined in 77% of the cases for calcium and vitamin D. Among frequent PPOs in patients with cardiovascular disease, the clinical relevance could not be determined in 21–33% of the cases when it came to treatment with angiotensin converting enzyme inhibitors, warfarin, beta-blockers, or statins.

In the main analysis, as well as the sensitivity analysis, the odds for ≥1 clinically relevant PIMs/PPOs were greater for patients with multidose drug dispensing and increased by the number of drugs in the medication list (Table 3). Age, sex, cognition, and nursing home residence was not statistically significantly associated with suboptimal drug treatment.

**Table 3:** Odds ratios (95% confidence interval) for ≥1 potentially inappropriate medications (PIMs) and/or ≥1 potential prescription omissions (PPOs), identified by the STOPP and START criteria, respectively, according to characteristics of the patient

	≥1 PIMs/PPOs according to			≥1 clinically relevant PIMs/PPOs according to		
	STOPP/START	STOPP	START	STOPP/START	STOPP	START
Age	1.05 (0.98; 1.12)	1.02 (0.97; 1.08)	1.02 (0.97; 10.7)	0.99 (0.93; 1.04) *1.04 (0.98; 1.10)*	1.01 (0.96; 1.06) *1.04 (0.98; 1.10)*	0.98 (0.93; 1.03) *1.03 (0.98; 1.08)*
Sex	0.73 (0.28; 1.92)	0.59 (0.27; 1.29)	0.81 (0.42; 1.58)	0.80 (0.37; 1.70) *0.72 (0.30; 1.73)*	1.24 (0.62; 2.46) *0.66 (0.31; 1.42)*	1.05 (0.55; 2.01) *0.97 (0.52; 1.80)*
MDD	10.6 (1.60; 70.1)	2.55 (0.87; 7.48)	2.23 (0.89; 5.60)	7.95 (2.35; 26.9) *6.96 (1.50; 32.3)*	2.90 (1.14; 7.38) *2.50 (0.88; 7.09)*	1.82 (0.77; 4.30) *2.01 (0.86; 4.72)*
Impaired cognition	1.28 (0.37; 4.40)	0.95 (0.37; 2.42)	1.52 (0.68; 3.39)	0.54 (0.20; 1.45) *1.13 (0.38; 3.38)*	0.69 (0.30; 1.59) *0.94 (0.38; 2.34)*	1.03 (0.48; 2.24) *1.23 (0.59; 2.58)*
Nursing home resident	0.29 (0.05; 1.77)	1.31 (0.44; 3.99)	0.46 (0.19; 1.16)	0.52 (0.17; 1.63) *0.22 (0.05; 1.00)*	1.17 (0.47; 2.89) *0.95 (0.33; 2.72)*	0.60 (0.27; 1.35) *0.34 (0.15; 0.76)*
Number of drugs	1.28 (1.11; 1.48)	1.35 (1.19; 1.53)	1.08 (0.99; 1.17)	1.28 (1.14; 1.43) *1.24 (1.09; 1.40)*	1.27 (1.15; 1.41) *1.37 (1.21; 1.54)*	1.08 (0.99; 1.17) *1.03 (0.96; 1.12)*

Figures in italics represent results in the sensitivity analysis where not assessable PIMs/PPOs were categorized as clinically relevant. Odds ratios which do not cross the line of unity are underlined

*STOPP* screening tool of older person’s potentially inappropriate prescription, *START* screening tool to alert to right treatment, *MDD* multi-dose drug dispensing

## Discussion

Our study shows that, at the overall level, one in two PIMs/PPOs, identified with the STOPP/START criteria, is clinically relevant according to the easily retrievable documentation. PIMs are clinically relevant to a greater extent, and PPOs are more often hard to assess concerning clinical relevance. The prevalence of over- and undertreatment according to various STOPP and START criteria varies, and for only six out of 65 STOPP and two out of 22 START criteria, a clinically relevant PIM and PPO, respectively, was identified in more than 5% of the patients.

In the scientific literature, there are numerous publications on the prevalence of suboptimal drug treatment according to various criteria [9]. In a review focusing specifically on the prevalence of STOPP/START criteria, the figures in various populations varied between 21 and 79% [10]. Our results are in the same range as these studies. Nevertheless, our finding that only half of the identified PIMs/PPOs were clearly clinically relevant at the individual level suggests that estimated prevalences of suboptimal drug treatment based on these criteria alone may need to be interpreted with caution.

As a first step to improve the quality of drug treatment in older people, without taking too much time and effort, physicians may want to consider to stop treatment with long-acting benzodiazepines and aspirin at doses >150 mg/day. Indeed, in this study, this kind of treatment could be subject to change in almost 100% of the cases. Similarly, the use of loop diuretics without clinical signs of heart failure and vasodilator drugs in patients with postural hypotension could be worth reconsideration. When it comes to identifying undertreatment and adding drug(s) to the medication list, it may be a successful strategy to start off to assess if a patient with cardiovascular disease would benefit from treatment with an angiotensin converting enzyme inhibitor, warfarin (or other oral anticoagulants), a beta-blocker, or a statin. Indeed, in seven to eight cases out of ten without such treatment, information available in the medical records will suffice to assess the need.

Regarding which patients for physicians to focus upon, those with multidose drug dispensing were to an increased extent subjected to suboptimal treatment. This finding supports previous studies where this system has been associated with fewer changes in drug treatment, and poorer quality of drug treatment [19–21]. Further, for those with many drugs, overtreatment needs to be considered. In fact, the number of drugs may reflect burden of disease [22], which, in turn, may suggest a greater treatment complexity. Regarding undertreatment, no significant predictor was identified in the main analysis, indicating that such treatment needs to be considered in all cases. Indeed, a previous study has shown that, at ≥2 drugs in the medication list, the number of missing drugs remains stable irrespective of the number of drugs in the medication list [23]. Interestingly, age and sex were not associated with clinically relevant PIMs/PPOs, although such an association has previously been shown regarding potentially relevant criteria [17]. Further, no significant association with cognition and nursing home residence was found.

In our study, 71% of identified PIMs and 32% of identified PPOs were considered clinically relevant. These figures are similar to the implementation rate reported in a recent study: 56 and 39% of PIMs and PPOs, respectively [24]. However, another study reported that 91% of STOPP and 97% of START recommendations were accepted by the attending physician [12]. The divergence between the results may be explained by the settings of the latter study. In that study, a research physician discussed recommendations based on STOPP/START with the attending medical team. Thus, PIMs/PPOs from the start assessed as not clinically relevant would probably not be discussed at all due to the medical expertise of the intervener, and therefore not captured in the denominator.

The results of this study may contribute to the understanding of the lack of effects for third party medication reviews regarding patient relevant outcomes reflecting the net effect of drug treatment, such as death and hospitalizations [25–27]. In addition to the fact that indicator sets may have a low sensitivity [28, 29], our study shows that only five in ten identified PIMs/PPOs were clinically relevant, and an additional three were hard to determine concerning clinical relevance given easily available information. The latter finding highlights the importance of a medical assessment of the entire patient as the basis to determine the appropriateness of drug treatment, including the medical history, a physical and/or psychiatric examination, and laboratory tests. Our findings may also explain that only a limited proportion of alerts to drug treatment changes upon third party medication reviews are acted upon [30].

The most important strengths of this study are that it provides knowledge on the clinical relevance of the STOPP/START criteria, and identifies easily manageable advice to improve drug treatment quality in older people. The results are strengthened by the fact that the underlying assessments of clinical relevance were performed by two specialist physicians with expertise in the relevant area. As could be expected, the inter-rater agreement was moderate, illustrating the subjectivity of clinical judgments and the advantage to involve two assessors.

The fact that we have focused on hip fracture patients may have implications for the generalizability of the results. However, these patients may represent a relevant subgroup of older patients since hip fracture is a common diagnosis in Sweden where every fourth middle-aged woman will sustain a hip fracture during her lifetime, and one out of three hip fracture patients is a man [31]. Further, suboptimal drug treatment is common in this patient group [32]. However, the prevalence of suboptimal drug treatment, especially inappropriate drugs related to fall risk, may differ from that found in a general population of older people, and the results may therefore mainly be applicable to hip fracture patients and frail older patients. Another limitation of this study is that the STOPP/START tools, which were used to systemize the specialist assessments, may not capture all kinds of suboptimal drug treatment [24].

In conclusion, this study illustrates the clinical relevance of the STOPP/START criteria sets, presenting suggestions on which criteria and which patients to focus upon for a physician in constraints of time. Indeed, a medical assessment is the key step when it comes to choosing drug treatment, as all prescribing has to be adapted to the characteristics of the individual patient.

## References

[1] Alldred DP, Kennedy MC, Hughes C, Chen TF, Miller P (2016). Interventions to optimise prescribing for older people in care homes. Cochrane Database Syst Rev.

[2] Gillaizeau F, Chan E, Trinquart L, Colombet I, Walton RT, Rege-Walther M, Burnand B, Durieux P (2013). Computerized advice on drug dosage to improve prescribing practice. Cochrane Database Syst Rev.

[3] Patterson SM, Cadogan CA, Kerse N, Cardwell CR, Bradley MC, Ryan C, Hughes C (2014). Interventions to improve the appropriate use of polypharmacy for older people. Cochrane Database Syst Rev.

[4] Laroche ML, Charmes JP, Merle L (2007). Potentially inappropriate medications in the elderly: a French consensus panel list. Eur J Clin Pharmacol.

[5] Holt S, Schmiedl S, Thurmann PA (2010). Potentially inappropriate medications in the elderly: the PRISCUS list. Dtsch Arztebl Int.

[6] American Geriatrics Society (2012). American Geriatrics Society updated beers criteria for potentially inappropriate medication use in older adults. J Am Geriatr Soc.

[7] Fastbom J, Johnell K (2015). National indicators for quality of drug therapy in older persons: the Swedish experience from the first 10 years. Drugs Aging.

[8] Gallagher P, Ryan C, Byrne S, Kennedy J, O'Mahony D (2008). STOPP (screening tool of older person's prescriptions) and START (screening tool to alert doctors to right treatment). Consensus validation. Int J Clin Pharmacol Ther.

[9] Tommelein E, Mehuys E, Petrovic M, Somers A, Colin P, Boussery K (2015). Potentially inappropriate prescribing in community-dwelling older people across Europe: a systematic literature review. Eur J Clin Pharmacol.

[10] Hill-Taylor B, Sketris I, Hayden J, Byrne S, O'Sullivan D, Christie R (2013). Application of the STOPP/START criteria: a systematic review of the prevalence of potentially inappropriate prescribing in older adults, and evidence of clinical, humanistic and economic impact. J Clin Pharm Ther.

[11] O'Mahony D, O'Sullivan D, Byrne S, O'Connor MN, Ryan C, Gallagher P (2015). STOPP/START criteria for potentially inappropriate prescribing in older people: version 2. Age Ageing.

[12] Gallagher PF, O'Connor MN, O'Mahony D (2011). Prevention of potentially inappropriate prescribing for elderly patients: a randomized controlled trial using STOPP/START criteria. Clin Pharmacol Ther.

[13] Dalleur O, Feron JM, Spinewine A (2014). Views of general practitioners on the use of STOPP&START in primary care: a qualitative study. Acta Clin Belg.

[14] Christensen T, Grimsmo A (2008). Instant availability of patient records, but diminished availability of patient information: a multi-method study of GP’s use of electronic patient records. BMC medical informatics and decision making.

[15] Andersen M (2006). Is it possible to measure prescribing quality using only prescription data?. Basic Clin Pharmacol Toxicol.

[16] Hoven JL, Haaijer-Ruskamp FM, Vander Stichele RH (2005). Indicators of prescribing quality in drug utilisation research: report of a European meeting (DURQUIM, 13–15 May 2004). Eur J Clin Pharmacol.

[17] Moriarty F, Bennett K, Fahey T, Kenny RA, Cahir C (2015). Longitudinal prevalence of potentially inappropriate medicines and potential prescribing omissions in a cohort of community-dwelling older people. Eur J Clin Pharmacol.

[18] Sjöberg C, Wallerstedt SM (2013). Effects of medication reviews performed by a physician on treatment with fracture-preventing and fall-risk-increasing drugs in older adults with hip fracture-a randomized controlled study. J Am Geriatr Soc.

[19] Sjöberg C, Edward C, Fastbom J, Johnell K, Landahl S, Narbro K, Wallerstedt SM (2011). Association between multi-dose drug dispensing and quality of drug treatment—a register-based study. PLoS One.

[20] Wallerstedt SM, Fastbom J, Johnell K, Sjöberg C, Landahl S, Sundström A (2013). Drug treatment in older people before and after the transition to a multi-dose drug dispensing system—a longitudinal analysis. PLoS One.

[21] Belfrage B, Koldestam A, Sjöberg C, Wallerstedt SM (2014). Prevalence of suboptimal drug treatment in patients with and without multidose drug dispensing—a cross-sectional study. Eur J Clin Pharmacol.

[22] Brilleman SL, Salisbury C (2013). Comparing measures of multimorbidity to predict outcomes in primary care: a cross sectional study. Fam Pract.

[23] Belfrage B, Koldestam A, Sjöberg C, Wallerstedt SM (2015). Number of drugs in the medication list as an indicator of prescribing quality: a validation study of polypharmacy indicators in older hip fracture patients. Eur J Clin Pharmacol.

[24] Verdoorn S, Kwint HF, Faber A, Gussekloo J, Bouvy ML (2015). Majority of drug-related problems identified during medication review are not associated with STOPP/START criteria. Eur J Clin Pharmacol.

[25] Christensen M, Lundh A (2016). Medication review in hospitalised patients to reduce morbidity and mortality. Cochrane Database Syst Rev.

[26] Hohl CM, Wickham ME, Sobolev B, Perry JJ, Sivilotti ML, Garrison S, Lang E, Brasher P, Doyle-Waters MM, Brar B, Rowe BH, Lexchin J, Holland R (2015). The effect of early in-hospital medication review on health outcomes: a systematic review. Br J Clin Pharmacol.

[27] Wallerstedt SM, Kindblom JM, Nylén K, Samuelsson O, Strandell A (2014). Medication reviews for nursing home residents to reduce mortality and hospitalization: systematic review and meta-analysis. Br J Clin Pharmacol.

[28] Wallerstedt SM, Belfrage B, Fastbom J (2015). Association between drug-specific indicators of prescribing quality and quality of drug treatment: a validation study. Pharmacoepidemiol Drug Saf.

[29] Wallerstedt SM, Belfrage B, Koldestam A, Sjöberg C (2016) Concurrent validity of Swedish drug-specific indicators of prescribing quality [Swedish] Läkartidningen 3

[30] Kwint HF, Bermingham L, Faber A, Gussekloo J, Bouvy ML (2013). The relationship between the extent of collaboration of general practitioners and pharmacists and the implementation of recommendations arising from medication review : a systematic review. Drugs Aging.

[31] Kanis JA, Johnell O, De Laet C, Jonsson B, Oden A, Ogelsby AK (2002). International variations in hip fracture probabilities: implications for risk assessment. J Bone Miner Res.

[32] Sjöberg C, Bladh L, Klintberg L, Mellström D, Ohlsson C, Wallerstedt SM (2010). Treatment with fall-risk-increasing and fracture-preventing drugs before and after a hip fracture: an observational study. Drugs Aging.

